# Theory-based strategies for teaching evidence-based practice to undergraduate health students: a systematic review

**DOI:** 10.1186/s12909-019-1698-4

**Published:** 2019-07-18

**Authors:** Mary-Anne Ramis, Anne Chang, Aaron Conway, David Lim, Judy Munday, Lisa Nissen

**Affiliations:** 1 Mater Health, Evidence in Practice Unit & Queensland Centre for Evidence Based Nursing and Midwifery, A Joanna Briggs Institute Centre of Excellence, South Brisbane, QLD 4101 Australia; 2 Queensland University of Technology, School of Nursing, Kelvin Grove Campus, Victoria Park Road, Brisbane, 4059 Australia; 3 Peter Munk Cardiac Centre, Toronto General Hospital, University Health Network, Lawrence S. Bloomberg Faculty of Nursing, University of Toronto, Toronto, ON M5G 2N2 Canada; 4 School of Science and Health, Western Sydney University, Sydney, 2751 Australia; 5 Queensland University of Technology, School of Clinical Sciences, Gardens Point Campus, QLD, Brisbane, 4000 Australia; 6 Queensland University of Technology, School of Nursing, Victoria Park Road, Kelvin Grove, Brisbane, Queensland 4059 Australia; 7 Faculty of Health and Sports Sciences, University of Agder, Grimstad, Norway

**Keywords:** Evidence-based practice, EBP, Undergraduate, Health professions, Education, Social cognitive theory, Theory-based intervention

## Abstract

**Background:**

Undergraduate students across health professions are required to be capable users of evidence in their clinical practice after graduation. Gaining the essential knowledge and clinical behaviors for evidence-based practice can be enhanced by theory-based strategies. Limited evidence exists on the effect of underpinning undergraduate EBP curricula with a theoretical framework to support EBP competence. A systematic review was conducted to determine the effectiveness of EBP teaching strategies for undergraduate students, with specific focus on efficacy of theory-based strategies.

**Methods:**

This review critically appraised and synthesized evidence on the effectiveness of EBP theory-based teaching strategies specifically for undergraduate health students on long or short-term change in multiple outcomes, including but not limited to, EBP knowledge and attitudes. PubMed, CINAHL, Scopus, ProQuest Health, ERIC, The Campbell Collaboration, PsycINFO were searched for published studies and The New York Academy of Medicine, ProQuest Dissertations and Mednar were searched for unpublished studies. Two independent reviewers assessed studies using the Joanna Briggs Institute Meta-Analysis of Statistics Assessment and Review Instrument.

**Results:**

Twenty-eight studies reporting EBP teaching strategies were initially selected for review with methodological quality ranging from low to high. Studies varied in course duration, timing of delivery, population and course content. Only five included papers reported alignment with, and detail of, one or more theoretical frameworks. Theories reported included Social Cognitive Theory (one study), Roger’s Diffusion of Innovation Theory (two studies) and Cognitive Apprenticeship Theory (one study). Cognitive Flexibility Theory and Cognitive Load Theory were discussed in two separate papers by the same authors. All but one study measured EBP knowledge. Mixed results were reported on EBP knowledge, attitudes and skills across the five studies.

**Conclusions:**

EBP programs for undergraduate health students require consideration of multiple domains, including clinical behaviors, attitudes and cognitive learning processes; Interventions grounded in theory were found to have a small but positive effect on EBP attitudes. The most effective theory for developing and supporting EBP capability is not able to be determined by this review therefore additional rigorous research is required.

**Electronic supplementary material:**

The online version of this article (10.1186/s12909-019-1698-4) contains supplementary material, which is available to authorized users.

## Background

Evidence-based practice (EBP) education is a recommended component of undergraduate health degree courses [1–3] aiming to provide students with a fundamental understanding and level of EBP capability upon graduation [4, 5]. The importance of effectively teaching EBP to health students to support requirements for professional licensing and/or registration is also emphasized in the literature [5–8]. EBP educational research to-date has historically focused on teaching EBP skills and knowledge to undergraduates, with lesser focus on EBP capability and/or long-term effect of learnt skills [9]. More specifically, despite recommendations to base EBP learning curricula on all the steps of the EBP process [5] many undergraduate programs focus on teaching for a level of competence in literature searching and appraisal skills, with less consideration of implementing and evaluating evidence in practice [10]. Programs that do address all components of the EBP process are challenging as they require students to integrate steps of the process with the conceptual model of EBP, namely the combination of best research evidence with clinical expertise and patient preference in order to provide optimal patient care [11, 12]. Other difficulties identified in regard to EBP curricula include timing of delivery of EBP interventions [7, 13], how to support student engagement with learning EBP [7, 14], level of clinical integration required for best learning outcomes [13] and most appropriate theoretical framework for underpinning EBP interventions to support and develop EBP behaviors [10, 15].

Several systematic reviews have been conducted on the effectiveness of strategies for teaching the EBP process to postgraduate students and/or clinicians [16–21]. Young, Rohwer, Volmink, and Clarke [6] synthesized 15 published and one unpublished systematic reviews, from 1993 to 2013, on EBP teaching strategies for a mixture of undergraduate and postgraduate student and health professional populations from medicine, nursing and allied health fields. Each included review evaluated single and/or multi-faceted educational interventions aimed at improving various EBP outcomes including, but not limited to, knowledge, critical appraisal skills, attitudes and EBP behaviors. Recommendations suggested teaching strategies should account for individual student factors such as learning style and capability as well as external organizational factors such as the setting of the learning activity and delivery format. The review suggested a combination of methods (e.g. journal clubs, small group discussions, incorporating clinical scenarios, lectures) had greatest effect on improving critical appraisal skills, EBP behaviors and knowledge [6].

A recent systematic review by Kyriakoulis et al. [7], suggests that while multi-faceted interventions may support undergraduate students learning about EBP, current evidence is insufficient to confidently determine which strategy is most effective. The review included 20 papers reporting use of EBP educational interventions in medicine, nursing, dentistry, pharmacy and allied health fields, suggested that multifaceted strategies including technology and /or simulation techniques, could influence undergraduate skills, knowledge and attitude towards EBP. Results indicated that the teaching strategies primarily focused on teaching information literacy skills (including critical appraisal), with few studies focusing on developing EBP implementation skills [7]. Additionally, difficulty in engaging students in learning about EBP was identified. Measures to address strategies for EBP engagement are crucial in academic and clinical environments to support students translating EBP competence to professional practice after graduation.

The challenge of implementing evidence in practice, across all health professions has led to recommendations for use of psychological and/or behavioral theory as an underpinning framework for implementation research and knowledge translation interventions [15, 22–26]. Theoretical constructs provide guidance for examining and understanding a concept in a manner that is generalizable, through aligning with prior work on how ideas can be organised and/or represented as well as regarding domains or dimensions of the concept being investigated [27, 28]. Such theoretically based interventions support extension beyond consideration of ‘what works best’ to address more in-depth understandings of why, how or when interventions may or may not be successful [29, 30]. The use of theory is recommended for complex interventions where behavior change is required [24, 29], or when trying to predict behavior change [31–33]. More specifically regarding EBP, some research exists incorporating Social Cognitive Theory (SCT) into interventions for promoting health professionals’ adoption of EBP, both in the clinical setting [28, 31, 34–36] and from an educational perspective [37]. Evidence also exists to support the predictive power of such theories [32, 33]. Eccles and colleagues suggest intention can be an acceptable measure for subsequent behavior in health professionals, when supported by an appropriate theoretical framework [31]. Undergraduate students’ intention to use EBP is influenced by a level of confidence and/or capability with the behaviors prior to graduation [4, 13, 38, 39], which is where theory-based programs may be effective. The question this systematic review addressed therefore, was, “What is the effectiveness of theory-based strategies aimed at teaching the EBP process to undergraduate health students?”

## Methods

### Modifications for the original protocol

The original protocol for this review was published on the Joanna Briggs Institute database [40] as well as on the PROSPERO register (CRD42015019032). Initially the review aimed to identify the overall effectiveness of EBP teaching strategies to undergraduate health students however prior to completion of our original review, another systematic review was published on this topic [7]. Considering the findings of that review, as well as other recent literature specifically on undergraduate EBP education for [10, 13, 39], a pragmatic decision was made to look critically at the selected studies to focus on those that reported underpinning their interventions in theory. Considering the potential impact theoretical constructs can have on behavior change [27, 31], as well as the association between student capability and their intention to use EBP after graduation [39], investigating any effect these types of interventions may have on student’s EBP skills, knowledge and other specified outcomes could identify strategies that further support EBP capability.

### Inclusion and exclusion criteria

For this review, an undergraduate student was defined as one who is completing their first formal university degree training for their particular discipline; however, it is acknowledged that there are some differences globally in teaching courses, nomenclature and durations for different health disciplines which may limit the synthesis of results. Included studies identified some or all of the five steps of the EBP process as outlined by Sackett et al. [12]. Experimental or comparative studies were considered for inclusion if they reported on any pedagogical and/or psychological theory as part of their intervention. As per our original protocol, outcomes of interest included EBP behavior, knowledge, skills, attitudes, self-efficacy (or self-confidence), beliefs, values and EBP use or future use.

### Search strategy

Databases searched include: PubMed, CINAHL, Scopus, ProQuest Health (including ProQuest Health and Medical Complete, ProQuest Nursing and Allied Health), ERIC, the Campbell Collaboration, PsycINFO and Science Direct. Unpublished studies were searched within The New York Academy of Medicine, ProQuest Dissertations and Mednar. Due to limited resources for translation, only studies published in English were sought. The initial search strategy, undertaken in July 2015 was updated in December 2016. Relevant published systematic reviews were hand searched [6, 7, 10, 20, 41] and any individual study that met inclusion criteria was retrieved. Published research arising from included dissertations was also sought. The initial search strategy for PubMed is included as (Additional file 1).

Two reviewers independently verified papers for inclusion and two independent reviewers assessed selected studies for methodological validity prior to final inclusion in the review, using the Joanna Briggs Institute Meta-Analysis of Statistics Assessment and Review Instruments (JBI-MAStARI) [42] for randomized controlled trials or one-group quasi-experimental studies, depending on study design. The instruments address risk of bias in specific aspects of the study methods, such as randomization, blinding, sampling and reporting. Any disagreements that arose between reviewers were resolved through consensus or with a third reviewer. Papers reporting educational interventions are known to be of varying and frequently low quality [43], therefore a minimum cut-off score of 3/10 was agreed upon for inclusion, however all papers that based their teaching strategy in theory were included for analysis in this review.

### Data extraction and synthesis

Two phases of data extraction were undertaken. Firstly, specific details were extracted of the intervention, geographical location, population, study design, methods and outcomes of significance to the review question and specific objectives, including details of the underpinning theory. Secondly, data extraction of the actual results of interventions, including statistical data was conducted. Heterogeneity in interventions, outcomes and outcome measurement tools, across and within studies, prevented meta-analysis, therefore a narrative and tabular analysis is presented.

## Results

### Description of studies and appraisal process

**Fig. 1:**
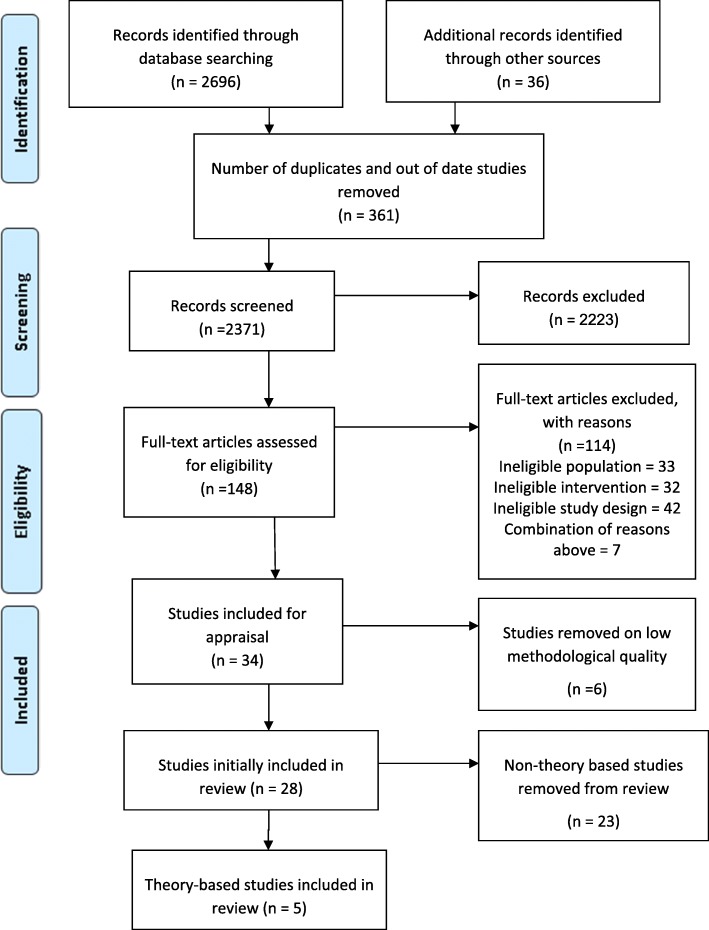
Literature search and study selection flow diagram: adapted from Moher et al. [44]

The initial search identified 2696 studies. A total of 2371 articles, titles and abstracts were examined, after removing duplicates, non-English and out of date range studies. From these, 148 full-text studies were retrieved. Reasons for exclusion at this stage were that the articles did not fit the systematic review criteria, for example they were not specific to undergraduate students, or were not empirical research studies. Verification of these studies by two reviewers (MR, EBA, ACh, ACo or DL) identified 34 published studies reporting on interventions for teaching EBP to undergraduates. These papers were assessed for quality with 28 being included initially. Reasons for the six studies being excluded at this stage included not addressing the outcomes of interest or insufficient statistical analysis. Following revision of the aim of the review (refer to Methods section), further examination of appraised studies identified five papers that based their teaching intervention on theory. These five studies became the primary focus of this paper with the aim of examining the effect of the theory-based teaching intervention on reported outcomes. A summary of the study details and components of the 23 non theory-based studies is attached as Additional file 2. The full search and selection process is outlined in the PRISMA [44] flowchart (Fig. 1). Risk of bias was identified across the five studies in areas regarding randomization, blinding and group allocation. Results of appraisal scores are presented in Additional file 3.

Two of the five included studies used quasi-experimental designs [45, 46], comparing their intervention to a control group who didn’t receive the intervention. Two other studies reported pre−/post-test results without a control group [47, 48] and the final study using a mixed-methods design [49]. The mixed-methods study comprised three study arms with quantitative and qualitative designs for testing their intervention, but one of these arms comprised post-registration doctoral students and was therefore not included in the analysis for this review [49]. Sample sizes ranged from 80 to 259 with a total of 933 participants. The studies included medicine, nursing, and nutrition students across different academic years. Overall, duration of the included interventions ranged from 10 sessions to 15 months and comprised techniques including didactic lectures, small group discussions, facilitated workshops and problem-based learning activities. Greater detail of the EBP interventions can be seen in Table 1.

**Table 1:** Components of theoretically-based EBP interventions for undergraduate students

				Steps of EBP process				
Citation detail	Discipline/ year level	Brief description of intervention	Duration of intervention	Question (PICO)	Searching	Critical Appraisal	Implementation	Evaluation of EBP (not intervention)
Ashktorab et al. 2014 [45]	Nursing; Final semester of degree course	Each step of intervention based on Rogers’ diffusion of Innovation Model; small groups; Q & A interactive discussions; encouraged to continue discussion on clinical questions outside of teaching hours; Posters presented at health centers as a way of sharing evidence at end of program.	Unclear; ten educational sessions delivered over course of unit but unit length not specified.	Yes – based on health priorities	Yes	Yes	Adoption of EBP behaviors	Not specified; evidence dissemination addressed in poster presentation
Kim et al. 2009 [46]	Nursing; Fourth year (senior year)	‘E-FIT’ intervention comprising 3 phases – 1) problem identification and evidence synthesis; 2) implementation strategy; 3) dissemination. 2-h introductory lecture on principles, definition, steps of and resources needed for EBP, Projects conducted in small groups; ended course with ‘Sharing Day’. Also included education on evidence dissemination strategies, protocol for practice change.	Conducted over full semester although specific time period not reported.	Yes	Yes, with librarian consult	Yes	Yes – protocol developed outlining steps to change practice; including analysis of organization, cost and SWOT analysis. Clinically integrated projects undertaken	Not specified; evidence dissemination addressed in sharing day with poster presentation
Liabsuetrakul et al., 2009 [47]	Fourth year medical students followed through to fifth year; examination at end of course (6th year)	Introductory session on fundamentals of EBM process then didactic lectures, small group work and facilitator guided sessions. Books, handouts, practice module used as course resources. Students paired with EBM mentors from different specialties. Group facilitators had facilitation manual.	Steps 1–3 of EBP process taught over 5 months in fourth year with didactic lectures (30 mins to 1 h) followed by relevant activities in small groups. Fifth year course commenced with revision lecture then lectures on steps 4 & 5; (total time for all 5 steps =15 months)	Yes	Yes	Yes	Theoretically and through observation of clinical staff practice to determine if practice was based on evidence	Yes
Liabsuetrakul et al., 2013 [48]	Fourth year medical students followed through to fifth year	Fourth years given clinical scenario: required to develop clinical questions, search for and appraise evidence for problem (e.g. diagnostic). Small group discussions - 5th year students: develop clinical questions from own clinical practice. Observation of clinicians undertaken to compare new knowledge with current practice. Facilitators had manual for guidance.	3 day short course at end of fourth year. After lectures, facilitated small group work undertaken. Intervention for fifth year as per previous paper. Total time for program = 10 months	Yes	Yes	Yes	Theoretically and through observation of clinical staff practice	Yes
Long et al., 2016 [49]	Arm 1 – Nursing (RN-BSN & MSN); Arm 2 – Nutrition (undergrad); Arm 3 – Pharmacy doctoral students; Academic years not specified	Evidence based internet research tool for teaching students across disciplines, about EBP (particularly critical appraisal skills). A 30-min training video was viewed by participants on how to use the tool. Case-study based questions included for students to apply question formulation and critical appraisal skills. Tool designed to be adjunct resource to texts and classroom education.	Unclear	Yes	Yes, with research librarian collaboration	Yes	Not specified	Not specified

### Findings of the review

#### Theories and intervention details presented in included studies

A brief description of the theories mentioned in the five included studies [45–49] is presented below, as well as detail on how the theory was addressed in relation to the intervention.

The intervention by Kim et al. [46] was reported to be based upon two theories: Bandura’s self-efficacy construct from SCT [50, 51] and Roger’s Theory of Diffusion of Innovations [52]. Bandura’s theory was addressed in the multiple regression modeling component of their study where students were asked to rate their confidence with making clinical decisions. Greater detail was presented regarding the second theory reported in this study - Rogers’ Theory of Diffusion of Innovations [52] - which proposes that new ideas can be built over time and through following a series of steps, be shared and adopted by others. One specific example of this as identified in the study was the use of an interactive assignment, which aligned with Rogers’ stage of adopting an innovation through social collaboration [46].

Ashktorab et al. [45] also grounded their intervention in Roger’s Theory of Diffusion Innovations [52] and clearly reported each stage of the intervention according to the five stages of Rogers’ theory. An example of how the knowledge acquisition phase was addressed was through provision of ten educational sessions with PowerPoint presentation and question and answer discussion sessions [45].

Long et al., [49] used Cognitive Apprenticeship Theory (CAT) for their EBP teaching strategy. This theory posits social interactions between the learner and the expert form a base for further cognitive development. Learning is accomplished through teaching techniques such as scaffolding, observation, modeling, mentoring, reflection and participation [53]. Such techniques gradually support learners and encourage them to delve even further into their learning experience. As part of the supplementary material for the paper, the authors included a hypothesized model of four elements of CAT (scaffolding, exploring, articulating, and reflecting) and strategies used to link the theory to the study intervention. For example, opportunities to practice skills were linked to the reflection component of the theory [49].

Liabsuetrakul et al. reported two studies, referring to Cognitive Load Theory (CLT) [54] in one study [48] and Cognitive Flexibility Theory (CFT) [55] in the other [47]. Although two different theories it was suggested in both studies that teaching techniques such as small group discussion, self-directed work and problem-based learning principles, along with integration of clinical scenarios, supported the theoretical principles, however attribution of individual techniques to specific elements of the proposed theories was not detailed.

### Outcomes

Reported outcome measures included EBM/EBP behaviors, knowledge, skills, attitudes, self-efficacy (or self-confidence), beliefs, values, EBP use or future use. A more detailed tabular summary of the statistical results is presented in Table 2.

**Table 2:** Study details for theory based EBP teaching strategies for undergraduate students

Study & country	Study design	Sample size	Outcome measures	Measurement scales	Measurement time point/s	Main results relative to systematic review
Ashktorab et al. 2014 Iran [45]	Quasi-Experimental with control	80 (control group n = 40; Intervention group, *n* = 40)	Knowledge, attitude, adoption	EBP questionnaire [56]	Before and after the intervention (paired data).	EBP Knowledge - Post intervention; significant mean difference between intervention (mean score 45.2, SD = 3.89) and control groups (mean score 31, SD = 7.05) (paired *t*-test, p < 0.0001). No significant difference in mean knowledge scores prior to intervention (control group mean = 30.3, SD = 5.26; Intervention group mean = 29.2, SD = 7.09; paired *t*-test, *p* = 0.43). EBP attitudes - intervention group showed greater improvement from baseline (pre-test mean score = 45.17, SD = 9.65; post-test mean score = 61.27, SD = 7.22): control group (pre-test mean score = 48.15, SD = 7.26; post-test mean score = 48.77, SD = 7.67) (independent *t* test, *p* < 0.0001
Kim et al. 2009 [46] USA	Quasi-Experimental pre-test, post-test study with control group	*N* = 208; intervention group = 88; control group = 120 Pre-test, post test data analyzed on 142 students (91 students competed pre and post data)	EBP knowledge, attitudes, use, future use	Johnston KAB questionnaire [57]	Beginning and end of semester (paired data).	(results all post intervention) EBP knowledge – small increase in intervention group mean = 5.68 (*n* = 65), SEM = 0.05; (*p* = 0.001) vs. control group mean = 5.43 (*n* = 72), SEM = 0.06; (p = 0.001). Mean difference = 0.25 (independent t-test, − 3.264; p = 0.001) EBP attitudes - no significant difference between control and intervention groups (mean diff = − 0.12, *p* = 0.398). EBP use - small significant increase in mean difference (mean diff = 0.26 (independent *t*-test, − 2.465, *p* = 0.015). EBP future use - no significant difference between groups (mean diff =0.13, *p* = 0.255). No significant differences at baseline.
Long et al. 2016 [49] USA & Lebanon	Mixed-methods with 3 arms to quant component; RCT/quasi-experimental;	Arm 1: N = 72 (USA/BSN); *N* = 23 (ME/BSN); *N* = 63 (USA/MSN); Arm 2: *N* = 37 (intervention); *N* = 21 (control); Arm 3: *N* = 31 (intervention); *N* = 39 (control)	Overall skills; application of skills; ability to distinguish credibility of information sources	Researcher developed assessment criteria based on tool by Ivanitskaya et al. [58] test, re-test reliability for Questions 1 & 2 (*r* = 0.83–0.81). Content validity testing reported 100% relevance to EBP	Pre–test at commencement of using tool; post-test within 3 weeks of completing assignment	EBP skills (web based intervention) - significant changes from baseline to follow up, for overall research skills in two different nursing undergraduate cohorts (*p* = 0.001) but no significant difference for distinguishing credibility of online sources (*p* = 0.070). Undergraduate students studying nutrition showed a significant positive difference between intervention and control groups from baseline to follow up. (*p* = 0.002) as well as significant difference from pre to post-test (*p* = 0.039).
Liabsuetrakul et al. 2009 [47] Thailand	Longitudinal one group pre-test, post-test;	*N* = 259	EBM attitudes, skill	Researcher developed tool (Cronbach’s Alpha > 0.85 for each item)	Before course, 5 months (T1) then 15 months after baseline (T2)	Significant increase in EBM attitudes from baseline: T0 to T1 (5 months) (*p* < 0.001) with a lesser but still significant effect (p < 0.001) from T1 to T2 (15 months after T0). EBM skills – mean scores improved from pre-test at both time points - 5 months and at 15 months
Liabsuetrakul et al. 2013 [49] Thailand	One group; pre-test, post- test;	*N* = 114	EBM knowledge, attitudes, skills	Researcher developed test; reliability analysis – Cronbach’s alpha 0.92	Before course, then at 1, 5, 13, 25 and 37 weeks post course (paired data)	EBM knowledge - increase in mean scores post intervention (*p* > 0.001) EBM skills: initial increase followed by a significant decrease in both groups when measured at weeks 5 and 13 (p < 0.001), increased significantly at 15 weeks (*p* = 0.05) after being given opportunity for individual learning and exposure to clinically scenarios. No significant difference between 4th and 5th year students (*p* = 0.17). EBM attitudes – 5th year students significantly lower mean score than 4th year students before intervention (p = 0.002). Linear modelling identified initial increase in scores, followed by decrease at second and third data collection points (weeks 1 & 5), with statistically significant increase 25 weeks after the original EBM course (*p* = 0.003). Authors suggest continuous teaching of EBP throughout the 5-year course may impact result.

#### EBP knowledge

EBP knowledge was measured in three of the included studies [45, 46, 48]. Small to moderate significant increases in EBP knowledge scores were reported in two studies of nursing students by Kim et al. [46] (mean difference = 0.25; *p* = 0.001) and Ashktorab et al. [45] (intervention group mean score 45.2, SD = 3.89; control group mean score 31, SD = 7.05; paired *t*-test, *p* < 0.0001). These scores were measured at completion of the intervention. Liabsuetrakul et al. [48] measured knowledge scores one week after completion of their intervention being delivered to medical students with an eight item summative assessment. Significant improvements were noted from pre-test scores to post-test (*p* < 0.001).

#### EBP attitudes

Four of the five studies measure EBP attitudes [45–48] with significant improvements noted in three of these studies [45, 47, 48]. Two studies measured immediate short-term changes in attitudes following their interventions [45, 46]. Ashktorab et al. [45] reported no significant difference in EBP attitudes, between control and intervention groups at baseline but a significant difference between groups after delivery of the EBP intervention to nursing students (*p* < 0.0001). Kim et al. reported no significant difference between groups for EBP attitudes (mean difference = 0.12; *p* = 0.398) with the authors suggesting delivering their intervention over a longer time may influence results. Liabsuetrakul et al. measured attitudes over a longer duration in both studies [47, 48]. A fluctuation of effect was noted with significant increase at week one (*p* < 0.001) [47, 48], followed by a slight decrease in scores at week five and week 13 but overall significant increase in scores from baseline at 37 weeks following the intervention (*p* = 0.007) [48]. Such results suggest time could be a factor for developing and/or sustaining positive EBP attitudes throughout the undergraduate curriculum.

#### EBP skills

Long et al. measured ‘overall research skills’ using a web-based tool that assessed searching and appraising evidence skills [49]. This measurement was recorded via self-report to a Likert scale question developed from the Research Readiness Self-Assessment tool [58]. Significant improvement from pre-test to post-test results was noted in nursing students using the tool (*p* = 0.001), as well as in the second arm of the study which was an RCT comprising intervention and control groups of undergraduate students studying nutrition (*p* = 0.002). Liabsuetrakul measured EBM skills in both studies [47, 48], through student self-reported answers to a Likert scale developed by the researchers. Fluctuations were again noted from baseline to different time points. Overall scores for EBM skills were significantly higher from baseline to week one (*p* < 0.001) [47, 48] and at 37 weeks post intervention (*p* = 0.003) [48], after students were given more time to reflect and conduct some individual learning.

#### EBP use and EBP future use

Only one study [46] measured outcomes of EBP use and EBP future use, using a validated tool developed by Johnston et al. [57]. The tool relies on student self-report but has high reliability and validity measures and has been tested in other studies of undergraduate students EBP [59–61]. A small but significant difference between intervention and control groups was reported for EBP use (mean difference = 0.26, *p* = 0.015), however no significant difference between groups was reported for EBP future use (mean diff =0.13, *p* = 0.255).

#### Other outcome measures

None of the included studies specifically measured outcomes of EBP self-efficacy, confidence or capability. Long et al. [49] measured an overall outcome of student ‘ability to distinguish credibility of online sources’, through measuring student responses to questions built into their web-based intervention. A non-significant difference (*p* = 0.70) between pre-test and post-test results as reported from the nursing arm of the study. In the second arm of the study, the nutrition students did report a significant difference between intervention and control groups after using the technology (*p* = 0.39). It was unclear if there were significant differences at baseline within or across the groups.

## Discussion

This systematic review aimed to identify effectiveness of theory-based interventions designed for improving undergraduate health students’ EBP. Effective learning of EBP requires consideration of cognitive, affective, behavioral and environmental elements [15], which is where theory-based interventions could be of value, however this review has identified that no single theory is yet aligned with EBP teaching and learning [15]. Due to heterogeneity in theories reported, populations and interventions it was not possible to confidently determine in this review which theory was most effective for effecting improvement in student EBP knowledge, skills, attitudes or other domains. However, the systematic review has identified some common elements influential to undergraduate EBP success in some domains, which require further exploration.

While each of the theories utilized in the studies had a different focus some overlapping concepts were noted. Social and environmental influences were noted in studies that used small groups and strategies for sharing evidence [45–47]. Such methods have been aligned with constructivism pedagogy and problem-based learning strategies [62, 63]. Learning is a social process [62] and for undergraduate students who are more now socially connected and technology aware, the power of social influence on successful learning must be considered [64]. Such influences are also recognized in, for example, Bandura’s Social Learning Theory (as a precursor to SCT) as affecting one’s self-efficacy to adopt certain behaviors [50]. EBP requires a level of cognitive ability as well as adoption of learnt behaviors therefore learning programs that acknowledge and accommodate social influences in both clinical and academic environments may be powerful to supporting students’ successful accomplishment of EBP skills.

Mixed results regarding changes in EBP knowledge were reported in the included studies. Only one included study measured EBP knowledge via a summative assessment [48] with other studies reporting short-term change in self-reported knowledge immediately following delivery of the EBP intervention. Measuring change in EBP knowledge has been a focal point of EBP interventions for many years with emphasis on the first three steps of the EBP process [6, 10, 65]. Undergraduate students require fundamental knowledge of these steps; however, without implementing strategies to improve students’ EBP attitudes and capability it may be that over time students feel less encouraged to use EBP in their respective clinical environments. Additional research monitoring changes over time and particularly on transition to professional practice is beyond the scope of this review but is suggested for future research.

The impact of role modeling on EBP behavior was acknowledged in three studies [46, 48, 49] in varying degrees and even though each of the studies included in the review used a different theoretical framework, there is growing support for consideration of role modeling in EBP education due to the positive impact on EBP beliefs and subsequent EBP behavior [66–68]. While role models in both academic and clinical areas are important, facilitators who can specifically support students with demonstrating how EBP knowledge learnt in the academic setting can be used in clinical contexts, have a critical role in EBP education across health disciplines [69–71]. Without seeing EBP in practice it can be difficult for undergraduate students across all disciplines to assimilate the components being taught and relevance to their future work.

The studies identified that students’ need time for reflection in order to assimilate their knowledge into practice and to develop positive EBP attitudes. The two studies reporting no significant difference in EBP attitudes [45, 46] were measured immediately after the intervention, while results from Liabsuetrakul et al. found improvement in EBP attitudes over time [47, 48]. Social psychology [72, 73] indicates that interventions for changing attitudes need to address affective, behavioral and cognitive components and that such change is more likely to occur in the longer rather than shorter term. EBP interventions targeting attitudes in the short-term are thus less likely to find significant improvement in attitude towards EBP as students require time to assimilate knowledge and influences from clinical and academic environments [69]. Teachings strategies incorporating regular feedback [69], opportunities to practice skills [69] and consideration of repeated or continuous strategies [7] have been suggested as ways to improve student engagement and facilitate sustained change.

Verbal persuasion (feedback), mastery of skills and vicarious experiences (role modelling) are three of the four sources of self-efficacy proposed by Bandura to promote self-efficacy for a specific task [50, 51]. SCT also proposes that individuals with higher self-efficacy for a specific activity will be more motivated to perform the activity [46, 50, 51]. While there is insufficient evidence in the systematic review to suggest SCT is the most effective theory for underpinning undergraduate EBP interventions, elements of the theory as discussed above have been reported in the literature [4, 66, 68] as well as the included studies [46, 48, 49] . Further consideration of these elements within teaching strategies for in EBP curricula is suggested for supporting student EBP self-efficacy and subsequent capability.

Synthesizing educational interventions presents many methodological challenges [74] and consequently there are several limitations to the review. Our initial search was targeted to find EBP teaching strategies for undergraduate students and retrieved a large number of papers which were carefully screened. It is unlikely but feasible that the decision to focus on the secondary aim of the review may have resulted in some specific theory-based papers being missed. Variation in international nomenclature for types of student and health professional categories is another limitation to the search process, as is the rapid expansion of studies being published in the field of EBP education. Although some repetition of reviews is acceptable for confirming results or uncovering different perspectives of a topic [75], following publication of recent reviews [7, 39], and advice from peer reviewers, we chose to focus on an aspect of the interventions which had not yet been addressed. We did not change the outcomes we were investigating, rather, synthesized the theoretical components of EBP educational interventions that were reported in studies obtained from our initial search. Modifications from original protocols are not uncommon [76, 77] however we acknowledge the impact this may have on certainty of the findings [76]. The review presents elements which can however, be explored further by EBP educators for supporting successful EBP learning and behavior adoption. A solid theoretical base provides a standardized platform for delivering an intervention, which can subsequently aid in maintaining intervention fidelity despite need for any contextual adaptations [30].

## Conclusion

EBP educational interventions for undergraduate health students are complex due to the cognitive and behavioral components necessary for success. Consequently, consideration of multiple domains, including clinical behaviors, attitudes and cognitive learning processes is required. Despite the requirements for undergraduate students to be capable EBP users after they graduate and the call for EBP education to be specific for the intended audience [37], the literature identifies limited theory-based evidence directed at undergraduate EBP education with a focus on preparing students to build capability and confidently use evidence in their professional practice.

Of the included studies, interventions grounded in theory were found to have a small but positive effect on EBP attitudes. Other common components were identified relating to time needed for learning as well as role modeling. Although this review was not able to determine the overall effect of these factors on specific outcomes due to heterogeneity in interventions, outcomes and measures, within and across the studies, such components require further investigation and their subsequent influence on EBP capability. Further research scoping the literature on undergraduate EBP curricula and underpinning theory is suggested.

## Additional Files

Additional File 1: Initial search strategy sample for PubMed database. (DOCX 13 kb)

Additional File 2: Summary of non theory-based studies selected for initial review. (DOCX 49 kb)

Additional File 3: Critical appraisal tables of all studies. (DOCX 14 kb)

## Data Availability

As this manuscript is a systematic review, all data generated or analyzed during this study are included in this published article or within the supplementary files. Further detail is available from the corresponding author on reasonable request.
