# Supplementary material for: Theory-based strategies for teaching evidence-based practice to undergraduate health students: a systematic review
Source: BMC Med Educ. 2019 Jul 18;19:267. doi: 10.1186/s12909-019-1698-4 (PMC6637485; doi:10.1186/s12909-019-1698-4)
Supplement: Supplementary file 1 — Initial search strategy sample for PubMed database. (DOCX 13 kb) [file 12909_2019_1698_MOESM1_ESM.docx]

**Supplementary file 1 - Search strategy sample – PubMed database**

**PubMed - July 2015; updated December 2016**

((((((((((((evidence-based practice[title/abstract]) OR evidence-based medicine[title/abstract] OR evidence-based practice [MeSH Major Topic] AND (Students, Health Occupations[MeSH Major topic] OR medical OR nursing OR physiotherapy OR (allied health) OR dentistry OR (occupational therapy) OR (speech Pathology) OR (social work) OR (physiotherapy) OR Psychology OR podiatry OR pharmacy) AND ((Critical appraisal) OR knowledge OR skills OR Attitude* OR self-efficacy OR self-confidence OR value* OR behavio* OR perception*) AND (Undergraduate[title/abstract]OR baccalaureate[title/abstract] OR college OR freshman OR student) AND ((Randomized controlled trial) OR (non-randomized controlled trial) OR RCT OR (quasi-experimental) OR Prospective OR retrospective OR(before and after) OR cohort stud* OR case-control* AND ( "2009/01/01"[PDat] : "2016/12/01"[PDat] ) AND English[lang]))))))))))))
