# Supplementary material for: Theory-based strategies for teaching evidence-based practice to undergraduate health students: a systematic review
Source: BMC Med Educ. 2019 Jul 18;19:267. doi: 10.1186/s12909-019-1698-4 (PMC6637485; doi:10.1186/s12909-019-1698-4)
Supplement: Supplementary file 2 — Summary of non theory-based studies selected for initial review. (DOCX 49 kb) [file 12909_2019_1698_MOESM2_ESM.docx]

| **Supplementary file 3: Summary of non-theory based studies initially selected for review** | | | | | | | |
| --- | --- | --- | --- | --- | --- | --- | --- |
| **Study & country** | **Study design** | **Sample size** | **Discipline/ academic year level** | **Outcome measures** | **Measurement scales** | **Measurement time point/s** | **Main results relative to systematic review** |
| Alahdab et al[[1](#_ENREF_1)]  Syria | One group; pre-test, post test | N=50 | Medicine; Mix of year levels; pre-clinical and clinical years | EBP knowledge, skills, attitudes and beliefs | Berlin tool [[2](#_ENREF_2)]  Plus own  questionnaire | Reported as before commencement of first session and at completion of last session but actual duration unclear. | **EBP knowledge** - Non-significant increase in Berlin test scores from pre-test to post-test = 2.68 (95% CI: 1.68-3.68; p>0.05); positive change in EBP attitudes & beliefs after course. |
| Aronoff et al [[3](#_ENREF_3)]  USA | One group; pre-test, post test | N=153 (n = 139 cases with paired data) | Medicine; Third year | EBM skills as per Fresno tool. | Fresno tool[[4](#_ENREF_4)] | Before and after the course (paired data). | **EBM skills** - Significant increase from pre-test result, following course: pre-course mean = 66.6; (95% CI = 46.5-86.7), post-test mean =77.7 (95% CI = 59-96.4) p<0.001. |
| Barghouti et al [[5](#_ENREF_5)]  Jordan | One group; pre-test, post test | N=54 | Medicine; Fifth year | Knowledge, skills | Fresno tool[[4](#_ENREF_4)] | First and last day of course (paired data). | Significant increase in **EBP knowledge and skills** reported (mean difference = 92.8, 95% CI: 84.7, 101.0, p< 0.001; effect size = 4.2) following intervention. |
| Bozzolan et al[[6](#_ENREF_6)]  Italy | Mixed methods study with quasi-experimental component comparing year levels | N=73  1^st^ yr, n = 26 2^nd^ year,  n = 28;  3^rd^ year,  n = 19) | Physio-therapy; First, second and third years | Knowledge, skills behavior (perceptions and attitudes explored with qualitative component of study) | A-Fresno (Italian version)[[7](#_ENREF_7)] audit of documents | Before and after each session, for each year group – period between testing was 205, 196 and 170 days for 1^st^, 2^nd^ and 3^rd^ years respectively. | **EBP knowledge** - Proportion of students passing test increased over academic year level, however within each year level, pre-test/post-test scores showed mixed results. Results assessing carrying-over of EBP knowledge showed no statistically significant change from first year to second year**. EBP behaviour** mixed results over year levels with identified challenges within clinical environment such as lack of role models and need for timely feedback.  Authors suggest reconsideration of minimum expected level of EBP training. |
| Cheng et al [[8](#_ENREF_8)]  Taiwan | RCT | N=94 (control group=47; intervention group = 47) | Medicine; Final year | Knowledge, behavior, attitudes, use (personal application) and future use | KAB questionnaire  [[9](#_ENREF_9)] | First and last day of the intervention (paired data). | Significant increase in all domains for before and after intervention scores (p<0.01) Across group results showed significant increase in intervention groups for **EBP knowledge** (p < 0.01) & **EBP use** (p < 0.001), non significant changes in EBP attitudes and EBP future use (p > 0.05). |
| Gagliardi et al[[10](#_ENREF_10)]  USA | Quasi-experimental | N = 30 | Medicine; Mixed third and fourth year | Attitudes, confidence, knowledge and skills | Berlin questionnaire [[2](#_ENREF_2)]  plus own developed survey for measuring confidence and attitudes | Immediately pre and post course measurements; 5 months post course follow up (T2) | **EBP knowledge and skills –** statistically significant median difference from pre-test to post test at T1 - reported as 13%  (Min 13%, max 73%; p<0.001). Significant improvements reported at T2 (n = 16) for some items regarding EBM attitudes and confidence (p = 0.002). |
| Ilic et al [[11](#_ENREF_11)]  Australia | RCT | N = 62 (intervention); N = 59 control group  6 month follow up N =60 (intervention); N = 37 (control) | Medicine; third year undergrad | EBM Skills (developing clinical question/ literature searching); self-perceived confidence | Fresno test (modified)[[4](#_ENREF_4)]  Clinical Effectiveness and Evidence-Based Practice questionnaire [[12](#_ENREF_12)] | 1 week post intervention; six months post intervention survey distributed regarding confidence in related skills. | No significant improvement in literature searching **EBM skills** between control and intervention groups (mean difference = 0.007; p = 0.99), despite students reporting increased confidence in literature searching skills and question development. Reinforcement of skills recommended during course. |
| Ilic et al[[13](#_ENREF_13)]  Australia and Malaysia | Mixed methods with RCT component | N=497; control group=74; intervention=73)  45 graduate entry; 102 under-  graduate entry | Medicine, third year (first clinical year) | EBM competency, skills and knowledge, self-efficacy, behaviors, attitudes | Berlin questionnaire [[2](#_ENREF_2)]  ACE questionnaire [[14](#_ENREF_14)]  EBPQ[[15](#_ENREF_15)] | One month following the intervention. | **EBM competency* -** no significant difference between Australian and Malaysian undergraduate students in either Berlin test or ACE questionnaire.  (Berlin Questionnaire: MD = -0.02; 95% CI -1.55, 1.51; p=0.89; ACE tool: mean rank difference = 13.12; p=0.09).  * data for other outcomes included graduate students |
| Jalali-Nia et al [[16](#_ENREF_16)]  Iran | One group; pre-test, post test | N=41  (Control group n=21; Intervention group, n=20) | Nursing; Second year | EBP knowledge, attitudes | Researcher developed questionnaires and MCQ tests. Reliability and validity for questionnaires (CVI >0.8/0.9); Cronbach’s alpha =0.8; 0.9 for attitude questionnaire) | Two open-ended questions asked before intervention; Questionnaires completed at end of semester for post intervention measures | No significant difference for **EBP knowledge** scores between groups (p>0.05, 95% CI: 20.07, 22.92); significant improvement in **EBP attitudes** for intervention group (p<0001, 95% CI: 13.27, 23.11).  [*Calculated Cohen’s *d* = 2.36 (95% CI: 1.5622, 3.157)] |
| Johnston et al [[17](#_ENREF_17)]  Hong Kong | Mixed methods with crossover design RCT | N=109 baseline to first assessment; N= 102 first assessment to 2^nd^ assessment | Medicine; Second year | EBM attitudes, knowledge, use and future use | Johnston KAB questionnaire [73] | Baseline, midpoint and at end of intervention. | Overall authors suggest intervention did not work for their context and a more traditional teaching format was preferred. As this was a cross over trial, both groups were ultimately exposed to both methods. |
| Khader et al[[18](#_ENREF_18)]  Jordan | One group before/after study | N = 256 (completed both questionnaires) | Medicine; Fourth year | EBM attitudes; EBM knowledge | Knowledge questionnaire developed by researchers based on previous study by McColl et al [[12](#_ENREF_12)] Attitudes measured with Likert scale | Before and after course | **EBM knowledge** - Increased proportion of students understanding concepts (p < 0.005).  **EBM attitudes** – “high proportion” of students reported high EBM attitudes before and after course. Recommendation for more longitudinal research. |
| Lai & Nalliah[[19](#_ENREF_19)]  Malaysia | One group pre-test, post-test | N =64 (N = 63 at follow up) | Medicine; Final six months of training | Behaviors and skills | Researcher developed questionnaire  (Cronbach’s alpha 0.84: 95% CI: 0.79-0.88) | Beginning and end of semester (6 months apart) | Significant improvements reported in students’ confidence in critical appraisal but negative impact on information seeking behaviours. |
| Lai and Teng[[20](#_ENREF_20)]  Malaysia | One group; pre-test, post test | N=72 | Medicine; Final year | EBM knowledge | Adapted Fresno  Test [[4](#_ENREF_4)] | Before and after training course (paired data). | **EBP knowledge** scores increased significantly from pre-test to post test results (p<0.001). Clinically integrated strategy resulted in greater improvements in EBM knowledge. |
| Leach et al [[21](#_ENREF_21)]  Australia | One group pre-test, post-test | N = 84 (group 1/pre-test); N = 33 (group 2/post-test) | Nursing; Third year | Attitudes toward EBP, EBP-related skills, Use of EBP, Barriers and Facilitators to EBP uptake | Evidence-based practice Attitude and utilization Survey (EBASE) developed by author [[22](#_ENREF_22)]  Cronbach alpha = 0.84 (CVI = 0.899) | Before and immediately after program (paired data). | Significant improvements in post-test **EBP skill** (Z =- 4.65; p < 0.001) and **EBP use** (Z = 3.12, p = 0.002) subscores but not **EBP attitudes** (Z = 1.08; p = 0.280). Statistically significant differences between groups at baseline were thoroughly reported (age, gender, previous health related work experience and years of health related work experience. |
| Ma et al [[23](#_ENREF_23)]  China | One group; pre-test, post test | N=251 | Medicine; Fifth year and eighth year | EBM knowledge, attitudes, use and future use | Johnston KAB questionnaire [[9](#_ENREF_9)] | Reported as before and after training sessions (paired data). | Improvements in all domains post-intervention: **EBM knowledge** - pre-intervention mean score of 21.57 ±2.99; post score total 25.59 ± 4.80 (p< 0.0001).  **EBM attitudes** - significant increase pre-test to post-test (12.91 ±5.55 vs. 10.08 ± 6.28; p < 0.0001)  **EBM behaviour** (personal use) - significant increase pre-test to post-test: 14.23 ±5.86 vs. 20.31 ±5.66; (p<0.0001). **EBM future use** - significant increase pre-test to post-test: 39.56 ±6.47; vs. 44.85 ±7.97 (p< 0.0001). |
| Nakagawa et al [[24](#_ENREF_24)]  Japan | One group; pre-test, post test | N = 37 | Pharmacy; Fifth year | Knowledge, readiness | Researcher developed questionnaire; 15 question EBM test | Immediately before and after workshop | Significant improvements in **EBM test scores (knowledge):** pre-test 11.4 ± 0.29 vs.post-test 12.6 ±0.22 (p < 0.0001). Levels of readiness and confidence significantly improved (p< 0.0001) but remained low. |
| Oh et al [[25](#_ENREF_25)]  Korea | One group; pre-test, post test | N=74 | Nursing; First year | EBP efficacy; barriers to research utilization | Researcher developed efficacy tool – Cronbach’s alpha 0.90 | Before and after EBP practical session (paired data). | **EBP efficacy** - statistically significant increase in overall confidence following the intervention (pre-test mean =2.30 (SD=0.35); post-test mean =3.05 (SD =0.38); mean diff = -0.75; p<0.001). |
| Ruzafa-Martinez et al[[26](#_ENREF_26)]  Spain | Quasi-experimental with control group | N = 59 (intervention group); N= 61 (control group) | Nursing; Second and third years | Attitude toward EBP; EBP knowledge; EBP skills & competence | EBP questionnaire developed by author [[27](#_ENREF_27)]  Cronbach’s alpha = 0.888 | Before and 2 months after 15 week intervention. | Significantly higher scores in intervention group at two months compared to control group, for all domains**.** |
| Sánchez-  Mendiola et al [[28](#_ENREF_28)]  Mexico | Quasi-  experimental parallel group controlled trial | N=289 | Medicine; Fourth, fifth, sixth years | EBM knowledge, attitudes and skills, use of resources | Taylor’s instrument [[29](#_ENREF_29)]Researcher developed MCQ for the study also – Cronbach’s alpha 0.82 | All outcomes measured at end of first semester, after course completed. | **EBM knowledge**: higher mean difference between 5^th^ year medical students completing EBM course, compared to 5^th^ year students who had not completed the course, for both Taylor’s instrument (Cohen’s *d* = 0.88, p<0.001) and MCQ (Cohen’s *d* = 3.54, p<0.001). Simultaneous validation -  6^th^ year students demonstrated statistically significant lower mean score than 5^th^ year intervention group in both assessments. **EBM attitudes:** 5^th^ year EBM group mean score significantly higher than 6^th^ year students completing course 12 months prior (p<0.05); significantly higher than 4^th^ year students yet to compete course (p<0.001). Effect size between 6^th^ year and 5^th^ year EBM group moderate (Cohen’s *d* =0.67*).* Despite slight drop in scores over time, authors report some sustained change in EBM attitudes. **Use of EBM resources:** significantly greater use of original research for clinical problems compared to control groups (p<0.01). |
| Umscheid et al [[30](#_ENREF_30)]  USA | Quasi-experimental; pre-test/post test | N = 117 (intervention group); N = 118 (control group) | Medicine; Second and third years | Competence, skills | Objective structured clinical exams (OSCE); questionnaire developed by researchers | End of year results compared | Use of clinically integrated EBM prescriptions improved **EBM competence** by 1.27 points overall (95% CI: 0.81, 1.72). Compared to observed structure clinical exams neither significant differences nor significant associations were reported regarding use of educational prescriptions and actual EBM behaviors. Students reported some benefit for aspects such as ‘acquiring’ or ‘appraising’ evidence. Those evaluating required greater awareness of current clinical practice. |
| West et al [[31](#_ENREF_31)]  USA | Quasi-experimental; longitudinal pre-test/post-test | Baseline 2^nd^ year N=65 (Fresno); N = 64 (Berlin): Baseline 3^rd^ year N = 64 (Fresno); N = 66 (Berlin)  Other sample sizes varied per item measured. | Medicine; second and third years | EBM knowledge and skills, attitudes | Fresno test [[4](#_ENREF_4)] Berlin questionnaire [[2](#_ENREF_2)] other non-validated, self-report items relating to EBM attitudes | Pre-test - first day of EBM course; post-test time point at end of second year after EBM course; second post-test measurement at point end of third year. | **EBM knowledge and skills:** Statistically significant improvements (p<0.001) at end of 2^nd^ and 3^rd^ years using both Berlin and Fresno tools. EBM assignment integrated into clinical rotation in 3^rd^ year. |
| Widyahening et al [[32](#_ENREF_32)]  Indonesia, Malaya, the Netherlands | Pre-test, post-test; three groups | N=526 in three groups, n= 224 (Indonesia- UI); n=202 (Malaya-UM); n=100 (Netherlands-UCMC) | Medicine; Third, fourth and sixth year | EBM knowledge, attitude, use, future use | Johnston KAB questionnaire [[9](#_ENREF_9)] | 26 items completed prior to course; only 11 items of knowledge/attitude  Scale completed at the end of the module (paired data - behavior not measured post intervention). | **EBM knowledge**: Two of the three universities reported significantly higher post-test knowledge scores when compared to their pre-test scores: UCMC– pre-test: mean score 5.04 (SD=0.40), post-test: mean score 5.35 (SD = 0.51) p<0.001; UM– pre-test: mean score 4.24 (SD=0.74), post-test mean score 4.53 (SD=0.72) p<0.001. The third university (UI) reported no significant change in post-test (p = 0.096)  **EBM attitudes:** post-test scores reported no change in any university, post-test. Authors report short term effects on knowledge but acknowledge contextual differences across universities contributed to varying results. **EBM use:** No significant differences between pre and post-test, across and within universities |
| Zhang et al [[33](#_ENREF_33)]  China | One group; pre-test, post test | N= 74 completed surveys | Nursing; year level not specified | EBP knowledge, attitudes, beliefs, behavior | Researchers developed own scale; reliability testing– Cronbach’s alpha 0.850 (attitudes); 0.897 (beliefs) 0.893 (behavior); 0.910 total | First survey completed after introductory session; post survey completed at end of final workshop discussion. | Significant improvements noted across all domains. **EBP knowledge:** Pre-intervention mean score of 11.51 ± 2.51, post-intervention score of 17.11 ± 3.30 (independent *t*-test, *t* = -11.64, p<0.001). **EBP attitudes & Beliefs:** Pre-intervention mean score of 35.67 ± 5.43 post-intervention score of 38.99 ± 4.04 (independent *t*-test, *t* = -4.24, p<0.001). **EBP Behaviour:** Pre-intervention mean score of 10.99 ± 2.77 post-intervention score of 15.32 ± 3.14 (independent *t*-test, *t* = -8.95, p<0.001). |

**References**

1. Alahdab F, Firwana B, Hasan R, Sonbol MB, Fares M, Alnahhas I, Sabouni A, Ferwana M: **Undergraduate medical students’ perceptions, attitudes, and competencies in evidence-based medicine (EBM), and their understanding of EBM reality in Syria**. *BMC research notes* 2012, **5**(1):431.

2. Fritsche L, Greenhalgh T, Falck-Ytter Y, Neumayer H, Kunz R: **Do short courses in evidence based medicine improve knowledge and skills? Validation of Berlin questionnaire and before and after study of courses in evidence based medicine**. *BMJ* 2002, **325**:1338 - 1341.

3. Aronoff SC, Evans B, Fleece D, Lyons P, Kaplan L, Rojas R: **Integrating evidence based medicine into undergraduate medical education: combining online instruction with clinical clerkships**. *Teaching and learning in medicine* 2010, **22**(3):219-223.

4. Ramos K, Schafer S, Tracz S: **Validation of the Fresno test of competence in evidence based medicine**. *BMJ (Clinical research ed)* 2003, **326**(7384):319.

5. Barghouti FF, Yassein NA, Jaber RM, Khader NJ, Shokhaibi SA, Almohtaseb A, AbuRmaileh N: **Short Course in Evidence-Based Medicine Improves Knowledge and Skills of Undergraduate Medical Students: A Before-and-After Study**. *Teaching and learning in medicine* 2013, **25**(3):191-194.

6. Bozzolan M, Simoni G, Balboni M, Fiorini F, Bombardi S, Bertin N, Da Roit M: **Undergraduate physiotherapy students' competencies, attitudes and perceptions after integrated educational pathways in evidence-based practice: a mixed methods study**. *Physiotherapy theory and practice* 2014, **30**(8):557-571.

7. Bozzolan M, Pavanello A, Barbieri R, Spada M, Del Giovane C, Gaiani R: **EDUCARE ALLA EVIDENCE BASED PRACTICE NELLA FORMAZIONE DI BASE: VALUTAZIONE DI ESITO CON IL TEST DI FRESNO ADATTATO (The adapted Fresno test to assess knowledge and skills in evidence based practice: The italian version)**. *Scienza Riabilitativa* 2012, **13**(2):12-18.

8. Cheng HM, Guo FR, Hsu TF, Chuang SY, Yen HT, Lee FY, Yang YY, Chen TL, Lee WS, Chuang CL: **Two strategies to intensify evidence-based medicine education of undergraduate students: a randomised controlled trial**. *Annals of the Academy of Medicine, Singapore* 2012, **41**(1):4-11.

9. Johnston JM, Leung GM, Fielding R, Tin KYK, Ho L: **The development and validation of a knowledge, attitude and behaviour questionnaire to assess undergraduate evidence-based practice teaching and learning**. *Medical Education* 2003, **37**(11):992-1000.

10. Gagliardi JP, Connie Schardt M: **Innovation in evidence-based medicine education and assessment: an interactive class for third-and fourth-year medical students**. *Journal of the Medical Library Association* 2012, **100**(4):306.

11. Ilic D, Tepper K, Misso M: **Teaching evidence-based medicine literature searching skills to medical students during the clinical years: a randomized controlled trial**. *Journal of the Medical Library Association: JMLA* 2012, **100**(3).

12. McColl A, Smith H, White P, Field J: **General practitioners' perceptions of the route to evidence based medicine: a questionnaire survey**. *Bmj* 1998, **316**(7128):361-365.

13. Ilic D, Hart W, Fiddes P, Misso M, Villanueva E: **Adopting a blended learning approach to teaching evidence based medicine: a mixed methods study**. *BMC medical education* 2013, **13**(1):169.

14. Ilic D, Nordin RB, Glasziou P, Tilson JK, Villanueva E: **Development and validation of the ACE tool: assessing medical trainees' competency in evidence based medicine**. *BMC medical education* 2014, **14**(1):114.

15. Upton D, Upton P: **Development of an evidence‐based practice questionnaire for nurses**. *Journal of advanced nursing* 2006, **53**(4):454-458.

16. Jalali‐Nia SF, Salsali M, Dehghan‐Nayeri N, Ebadi A: **Effect of evidence‐based education on Iranian nursing students' knowledge and attitude**. *Nursing & health sciences* 2011, **13**(2):221-227.

17. Johnston JM, Schooling C, Leung GM: **A randomised-controlled trial of two educational modes for undergraduate evidence-based medicine learning in Asia**. *BMC medical education* 2009, **9**(1):1.

18. Khader YS, Batayha W, Al‐Omari M: **The effect of evidence‐based medicine (EBM) training seminars on the knowledge and attitudes of medical students towards EBM**. *Journal of evaluation in clinical practice* 2011, **17**(4):640-643.

19. Lai NM, Teng CL: **Competence in evidence-based medicine of senior medical students following a clinically integrated training programme**. *Hong Kong Med J* 2009, **15**(5):332-338.

20. Lai NM, Teng CL: **Self-perceived competence correlates poorly with objectively measured competence in evidence based medicine among medical students**. *BMC Med Educ* 2011, **11**:25.

21. Leach MJ, Hofmeyer A, Bobridge A: **The impact of research education on student nurse attitude, skill and uptake of evidence‐based practice: a descriptive longitudinal survey**. *Journal of clinical nursing* 2016, **25**(1-2):194-203.

22. Leach MJ, Gillham D: **Evaluation of the Evidence‐Based practice Attitude and utilization SurvEy for complementary and alternative medicine practitioners**. *Journal of evaluation in clinical practice* 2008, **14**(5):792-798.

23. Ma X, Xu B, Liu Q, Zhang Y, Xiong H, Li Y: **Effectiveness of evidence-based medicine training for undergraduate students at a Chinese Military Medical University: a self-controlled trial.** *BMC Med Educ,* 2014, **14**(133).

24. Nakagawa N, Murai Y, Yoshida M, Suzuki H, Mano N: **Effects of an evidence-based medicine workshop on Japanese pharmacy students’ awareness regarding the importance of reading current clinical literature**. *Journal of pharmaceutical health care and sciences* 2015, **1**(1):23.

25. Oh EG, Kim S, Kim SS, Kim S, Cho EY, Yoo JS, Kim HS, Lee JH, You MA, Lee H: **Integrating evidence-based practice into RN-to-BSN clinical nursing education**. *The Journal of nursing education* 2010, **49**(7):387-392.

26. Ruzafa-Martínez M, López-Iborra L, Barranco DA, Ramos-Morcillo AJ: **Effectiveness of an evidence-based practice (EBP) course on the EBP competence of undergraduate nursing students: A quasi-experimental study**. *Nurse education today* 2016, **38**:82-87.

27. Ruzafa-Martinez M, Lopez-Iborra L, Moreno-Casbas T, Madrigal-Torres M: **Development and validation of the competence in evidence based practice questionnaire (EBP-COQ) among nursing students**. *BMC medical education* 2013, **13**(1):19.

28. Sánchez-Mendiola M, Kieffer-Escobar LF, Marín-Beltrán S, Downing SM, Schwartz A: **Teaching of evidence-based medicine to medical students in Mexico: a randomized controlled trial**. *BMC medical education* 2012, **12**(1):107.

29. Taylor R, Reeves B, Mears R, Keast J, Binns S, Ewings P, Khan K: **Development and validation of a questionnaire to evaluate the effectiveness of evidence-based practice teaching**. *Medical Education* 2001, **35**(6):544-547.

30. Umscheid CA, Maenner MJ, Mull N, Veesenmeyer AF, Farrar JT, Goldfarb S, Morrison G, Albanese MA, Frohna JG, Feldstein DA: **Using educational prescriptions to teach medical students evidence-based medicine**. *Medical teacher* 2016:1-6.

31. West CP, Jaeger TM, McDonald FS: **Extended evaluation of a longitudinal medical school evidence-based medicine curriculum**. *Journal of general internal medicine* 2011, **26**(6):611-615.

32. Widyahening IS, van der Heijden GJ, Moy FM, van der Graaf Y, Sastroasmoro S, Bulgiba A: **Direct short-term effects of EBP teaching: change in knowledge, not in attitude; a cross-cultural comparison among students from European and Asian medical schools**. *Medical education online* 2012, **17**.

33. Zhang Q, Zeng T, Chen Y, Li X: **Assisting undergraduate nursing students to learn evidence-based practice through self-directed learning and workshop strategies during clinical practicum**. *Nurse Education Today* 2012, **32**(5):570-575.
