# Supplementary material for: Theory-based strategies for teaching evidence-based practice to undergraduate health students: a systematic review
Source: BMC Med Educ. 2019 Jul 18;19:267. doi: 10.1186/s12909-019-1698-4 (PMC6637485; doi:10.1186/s12909-019-1698-4)
Supplement: Supplementary file 3 — Critical appraisal tables of all studies. (DOCX 14 kb) [file 12909_2019_1698_MOESM3_ESM.docx]

| **Assessment of methodological quality of RCT/Quasi-experimental theory-based studies** | | | | | | | | | | |
| --- | --- | --- | --- | --- | --- | --- | --- | --- | --- | --- |
| **Citation** | **Q1** | **Q2** | **Q3** | **Q4** | **Q5** | **Q6** | **Q7** | **Q8** | **Q9** | **Q10** |
| Kim et al. 2009 | N | U | U | N | U | Y | Y | Y | Y | Y |
| Long et al. 2016 | Y | U | U | N | U | U | Y | Y | U | Y |
| Ashkotorab et al. 2014 | U | U | U | U | U | Y | Y | Y | Y | Y |
| **Total** | 33.3% | 0 | 0 | 0 | 0 | 66.6% | 100% | 100% | 66% | 100% |

**Assessment of methodological quality of included one-group, pre-test/post-test theory-based studies (max possible score 6/10)**

| **Citation** | **Q1** | **Q2** | **Q3** | **Q4** | **Q5** | **Q6** | **Q7** | **Q8** | **Q9** | **Q10** |
| --- | --- | --- | --- | --- | --- | --- | --- | --- | --- | --- |
| Liabsuetrakul et al. 2009 | NA | NA | NA | NA | U | NA | NA | Y | Y | Y |
| Liabsuetrakul al. 2013 | NA | N | NA | N | N | NA | NA | Y | Y | Y |
| **Total** | 0 | 0 | 0 | 0 | 0 | 0 | 0 | 100% | 100% | 100% |

Questions from JBI Critical appraisal checklist for Randomised control/pseudo-randomised Trial: 1.Was the assignment to treatment groups truly random? 2. Were participants blinded to treatment allocation? 3. Was allocation to treatment groups concealed from the allocator? 4. Were the outcomes of people who withdrew described and included in the analysis? 5. Were those assessing outcomes blind to treatment allocation? 6. Were the control and treatment groups comparable at entry? 7. Were groups treated identically other than named interventions? 8. Were outcomes measured the same way for all groups? 9. Were outcomes measured in a reliable way? 10. Was appropriate statistical analysis used?

Appraisal tool source: The Joanna Briggs Institute, 2014. Joanna Briggs Institute Reviewer’s Manual: 2014 edition, Adelaide. ISBN: 978-1-920684-11-2

(Note: updated critical appraisals tools have been developed since completion of this review, available from: <http://joannabriggs.org/research/critical-appraisal-tools.html>)

Results of methodological appraisal of non-theory based studies available on request
